# PrIntMap-R: An Online Application for Intraprotein Intensity and Peptide Visualization from Bottom-Up Proteomics

**DOI:** 10.1021/acs.jproteome.2c00606

**Published:** 2023-01-18

**Authors:** Simon D. Weaver, Christine M. DeRosa, Sadie R. Schultz, Matthew M. Champion

**Affiliations:** † Department of Chemistry and Biochemistry, University of Notre Dame, Notre Dame, Indiana 46556, United States; ‡ Integrated Biomedical Sciences Graduate Program, University of Notre Dame, Notre Dame, Indiana 46556, United States

**Keywords:** hydrogen−deuterium exchange, glycosylation, post-translational modification, sequence coverage, visualization, domain-mapping quantitative sequence coverage

## Abstract

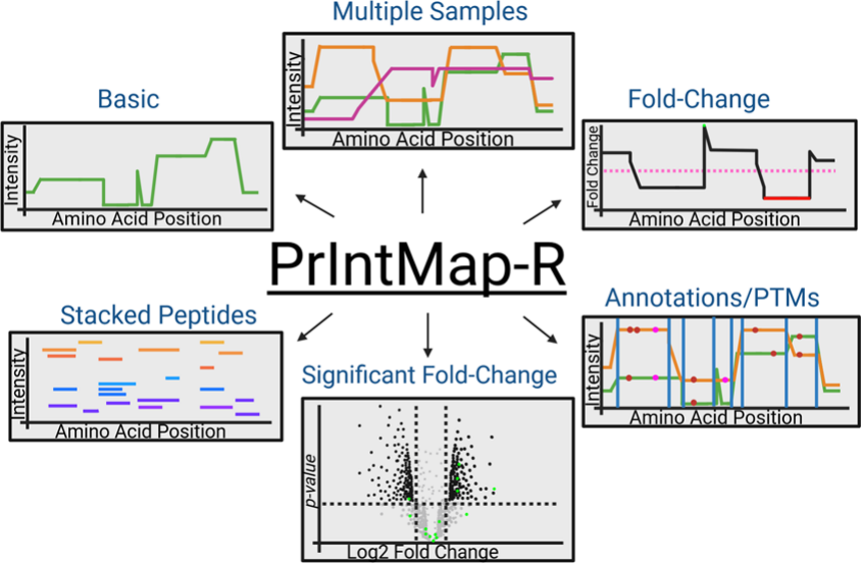

Bottom-up proteomics (BUP) produces rich data, but visualization and analysis are time-consuming and often require programming skills. Many tools analyze these data at the proteome-level, but fewer options exist for individual proteins. Sequence coverage maps are common, but do not proportion peptide intensity. Abundance-based visualization of sequence coverage facilitates detection of protein isoforms, domains, potential truncation sites, peptide “hot-spots”, and localization of post-translational modifications (PTMs). Redundant stacked-sequence coverage is an important tool in designing hydrogen–deuterium exchange (HDX) experiments. Visualization tools often lack graphical and tabular-export of processed data which complicates publication of results. Quantitative peptide abundance across amino acid sequences is an essential and missing tool in proteomics toolkits. Here we created PrIntMap-R, an online application that only requires peptide files from a database search and FASTA protein sequences. PrIntMap-R produces a variety of plots for quantitative visualization of coverage; annotation of specific sequences, PTM’s, and comparisons of one or many samples overlaid with calculated fold-change or several intensity metrics. We show use-cases including protein phosphorylation, identification of glycosylation, and the optimization of digestion conditions for HDX experiments. PrIntMap-R is freely available, open source, and can run online with no installation, or locally by downloading source code from GitHub.

## Introduction

Bottom-up proteomics (BUP) is a powerful analytical technique that generally involves the proteolytic digestion of simple or complex protein samples. Subsequent analysis of the resulting peptides is typically performed with tandem mass spectrometry (MS/MS) after the separation and ionization by capillary electrophoresis or nano liquid chromatography coupled to electrospray ionization.^1,2^ The raw mass spectrometry from modern hybrid instruments (MS) files can be very large, and contain tens of thousands of MS^1^ and MS/MS spectra. These spectra are assigned to the peptides from which they originated by database searching, peptide spectral mass matching, and protein inference.^3−5^ This is an informatics-heavy process where fragment ions are matched to probable peptide fragments, and aggregated into proteins via inference. There are multiple softwares available for database searching, each of which exports the identified peptide and protein data in slightly different formats, often as either .csv or .tsv files.^3,4,6^ Most of these software tools also contain built-in graphical interfaces; however, it is not straightforward to determine which tool if any exist within that framework to perform quantitative protein-level mapping with respect to amino-acid positions. PEAKS for example (Table 1) displays a heat bar underneath the sequence coverage which is proportional to PSMs assigned to that sequence, but not intensity or label-free quantitative (LFQ) abundance.^7^ Most search engines also lack adequate export figures from quantitative data, necessitating researchers to employ “screen-shots” as the lowest barrier to visualize data for publication.^7,8^

**Table 1:** Proteomic Data Visualization Tools Comparison

Tool name	Compatible with all major operating systems	Freely available	Accepted user data import file types	Includes PTM annotations	Multiple sample comparison (of protein coverage)	Determines uniqueness of peptides	Provides protein coverage visualization	Provides quantitative protein coverage visualization	Includes specific sequence annotations (i.e., protease cut sites)	Provides visualization of statistical significance (volcano plot)	Reference
PrIntMap-R	Yes	Yes	PEAKS, MSFragger, MaxQuant, MetaMorpheus, Proteome Discoverer, and generic .csv output files	Yes (for PEAKS and generic file formats)	Yes	Yes	Yes	Yes	Yes	Yes	NA
Skyline	No	Yes	MS files	Yes	Yes	Yes	Yes	No	No	Yes	MacLean, 2010^14,15^
Proteome Discoverer	No	No	Thermo .raw files	Yes	Yes	Yes	Yes	No	No	Yes	Orsburn, 2021^16^
Protease Guru	No	Yes	Proteome Databases	Yes	No	Yes (per protease not per protein)	Yes	No	Yes	No	Miller, 2021^17^
PEAKS	Yes	No	MS files	Yes	Yes	Yes	Yes	No	No	No	Tran, 2019^7^
piNET	Yes	Yes	List of peptide sequences, MaxQuant .txt output file, or generic .csv file	Yes	No	Yes	No	No	No	Yes	Shamsaei, 2020^18^
Peptigram	Yes	Yes	MaxQuant. txt output or generic .csv file	Yes	Yes	No	Yes	Yes	Yes	No	Manguy, 2017^19^
Peptide Shaker	Yes	Yes	MS files	Yes	No	No	Yes	No	No	No	Farag, 2021^11^
PSpecteR	Yes	Yes	MS/MS file (mzML, mzXML, or ThermoFisher raw)	Yes	No	No	Yes	No	No	No	Degnan, 2021^20^
Sequence Coverage Visualizer	Yes	Yes	List of peptide sequences	Yes	No	No	Yes	No	No	No	Shao, 2022^21^
Perseus	Win	Yes	MaxQuant and generic .csv	Yes	Yes	Yes	No	Yes per proteome	User and Plug-in options	Yes	Tyanova 2016^9^
Scaffold	Yes	No (free viewer)	Most (i.e. .PD, .DAT, MS-Fragger, generic, ...	Yes	Some	Yes (modular)	Some	Yes per proteome	Yes (modular)	Yes (modular)	Searle 2010^10^

In addition to database search software, there is a robust collection of resources to help analyze, visualize, and interpret outputs of these searches, many of which are summarized in Table 1. Additionally, programs like Microsoft Excel provide a platform with which to perform simple analyses with database search outputs in the form of spreadsheets. This has the advantage of ease-of-entry and portability, but lacks from repeatability (scripting) and transparency (“code” is not stand-alone). Many of the existing tools are designed for statistical analysis and visualization at the proteome level.^9−12^ Additionally, data reduction with programming languages such as R, Python, and Matlab is often required for more nuanced and targeted analysis.^13^ There is a need for versatile visualization tools at the protein level, for investigating specific proteins and how peptide coverage compares across domains and experimental conditions. For this purpose, we present PrIntMap-R (Protein Intensity Mapper), an online app written in R, which can also be downloaded and run locally.

PrIntMap-R includes numerous visualization features for intraprotein and peptide intensity in BUP experiments. A comparison of features between PrIntMap-R and other protein visualization tools is provided in Table 1, with a focus on the visualization aspects of the programs. Peptigram^19^ is a robust tool with several similar features to the PrIntMap-R, including being one of the two tools presented that provides some level of quantitative visualization of protein coverage. Peptigram incorporates a heatmap coloring gradient to capture intensity along their protein profile plots; however, here we compute protein coverage by plotting various summed or averaged intensity values against their corresponding residue in a protein of interest, making amino acid visualization possible, and providing multiple different methods to view the intensity. Perseus in specific might be the most robust implementation of these tools, but it is thought to have a steep learning curve.^9,10^

Additionally, PrIntMap-R is equipped with unique coverage comparison tools for multiple samples, including fold-change and difference representations of intensity values to quantify coverage change across samples/replicates. PrIntMap-R is also one of five tools from Table 1 that include specific sequence annotations, such as protease cleavage sites, on protein coverage maps, with our tool allowing users to specify any custom amino acid sequence of interest. Importantly, we combine our quantitative coverage visualization with sequence and PTM annotations, as well as multiple sample views so that many variables important in proteomic data visualization can be distilled into one plot. An additional feature unique to PrIntMap-R is the ability to accept user-generated peptide input files from at least five major database searching software packages, including the option for a generic .csv file independent of any search software. PrIntMap-R provides both a browser-enabled interface and stand-alone operation to facilitate the user experience. PrIntMap-R provides a combination of tools and a user-friendly interface with increased versatility, and is a valuable contribution to the available options for BUP data analysis and visualization.

## Methods

### The Tool

PrIntMap-R is a *shiny* app written in R, and available both online (https://championlab.shinyapps.io/printmap-r/) and as a downloadable package that can be run locally.^13,22^ The offline version requires only that R is installed on the computer, along with freely available packages, and works with both Mac and Windows operating systems. PrIntMap-R is fully open source; and everything required to run the app is free and available for download. Instructions for local installation and the app download can be found at https://github.com/Champion-Lab/PrIntMap-R, along with the source code.

**Figure 1:**
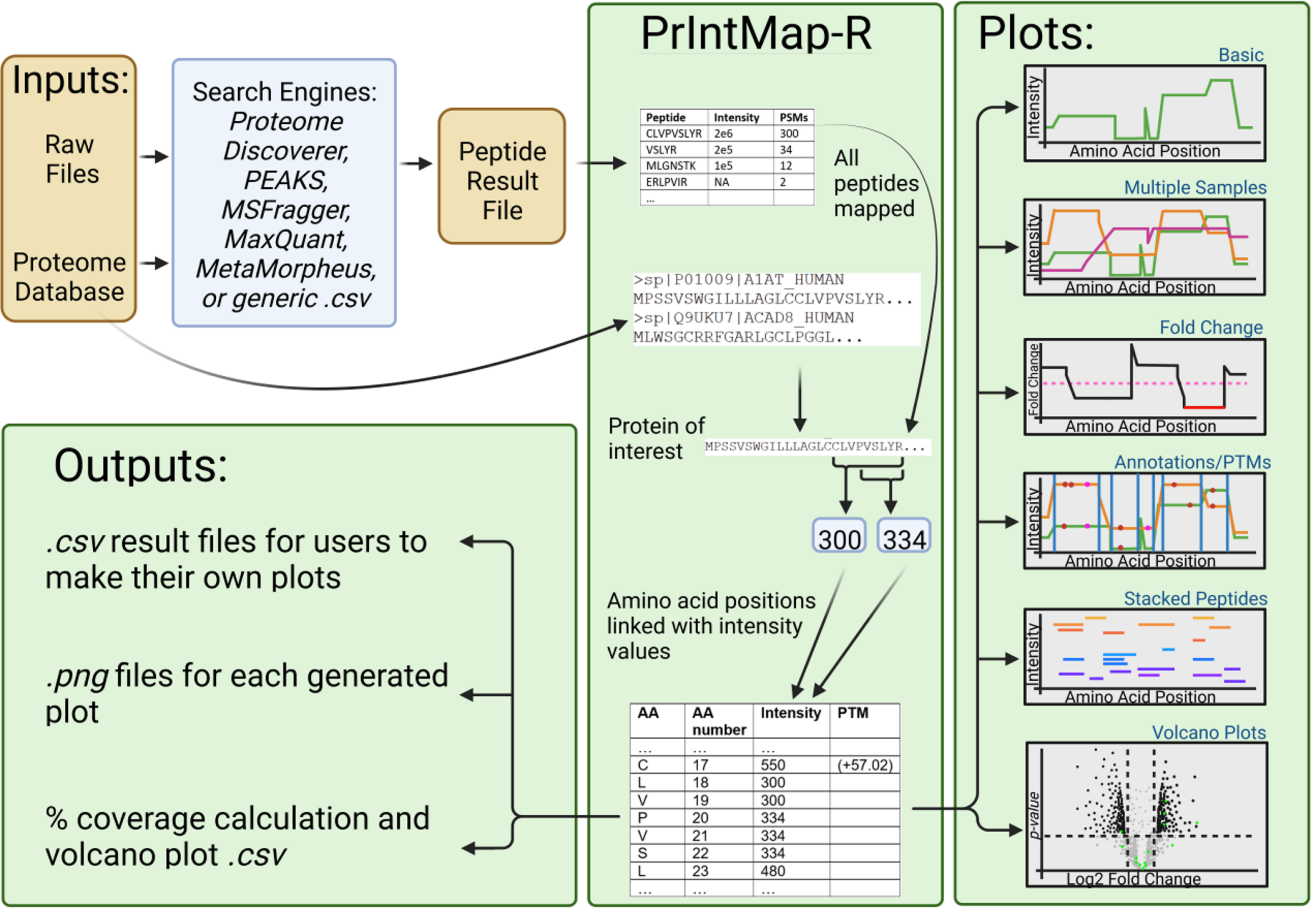
Data flowchart for PrIntMap-R. PrIntMap-R takes the peptide output files from one of several database search programs, and along with a FASTA protein database, constructs many plots showing various aspects of peptide coverage in a quantitative format, with options to compare multiple samples, annotate specific sequences and PTMs, as well as show stacked individual peptides. PrIntMap-R also lets the user download the raw data used to create each plot so users can easily create figures in the program of their choice.

The app takes peptide files that result from database searches, along with a FASTA protein sequence file, and renders a variety of plots visualizing peptide intensity across the amino acid sequence of proteins of interest (Figure 1). In addition to direct compatibility with PEAKS, MSFragger, Metamorpheus, MaxQuant, and Proteome Discoverer, PrIntMap-R is also compatible with a generic .csv file format as input, allowing the user to construct their own peptide file(s). Briefly, all peptides are mapped to the protein sequence, and peptide matches are stored in a dataframe. Each amino acid position is assigned an intensity value based on the sum of the total peptide intensities, spectra, or LFQ covering that position, and the intensity metric is determined by user input, where options include peptide spectral matches (PSMs), intensity (usually a fractional measure of total ion current), and peak area. LFQ data from PEAKS, MSFragger, Metamorpheus, Proteome Discoverer, and MaxQuant is interpreted as area values. PrIntMap-R is able to take combined peptide files with many experimental conditions in one file, as well as individual sample files. Combined files are parsed based on user input RegEx patterns, which allows for the combination of biological or technical replicates, the isolation of particular experimental conditions, or even the isolation of particular injections of interest. Options include summing or averaging replicates.

**Figure 2:**
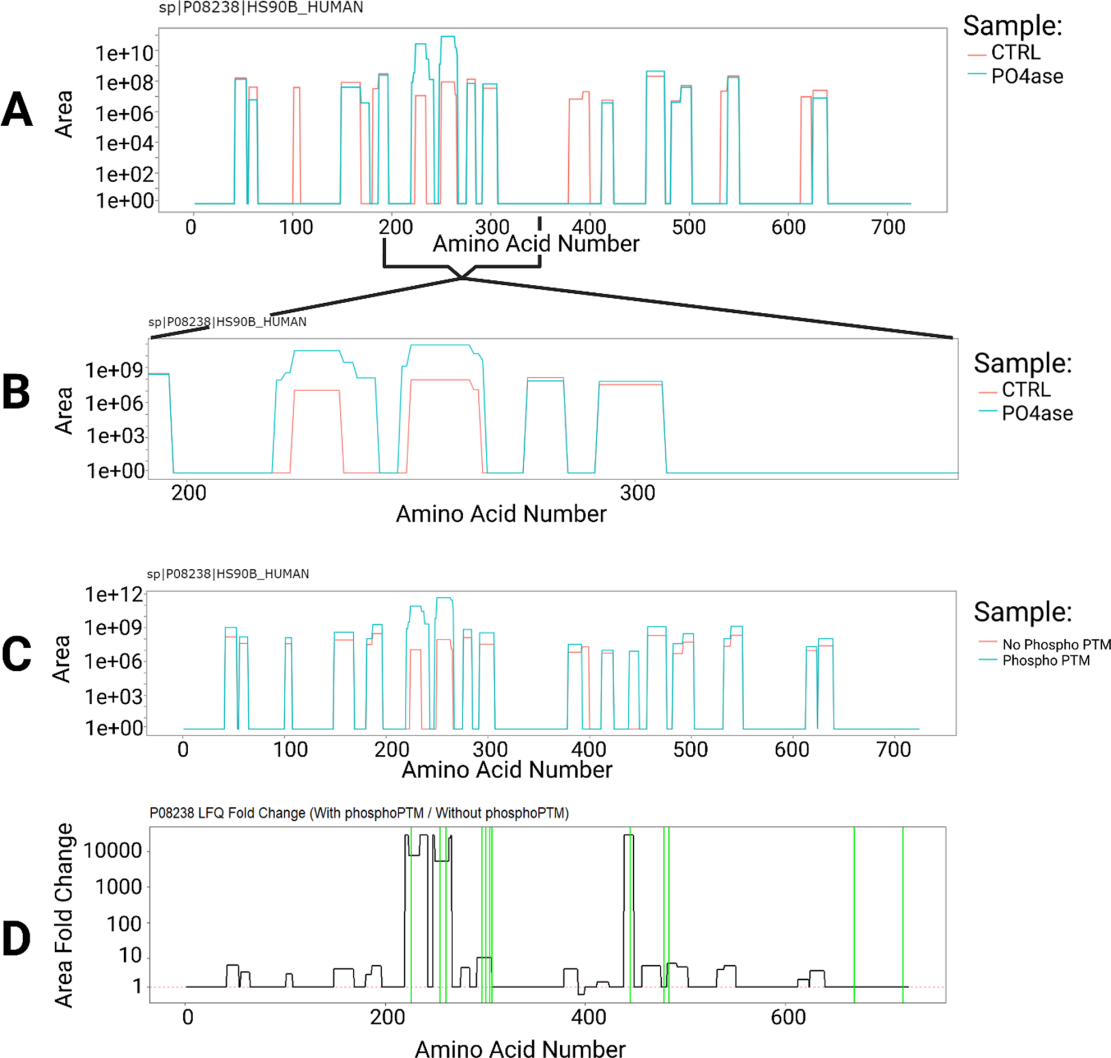
Phosphorylation analysis of heat shock protein HSP 90-beta (P08238) from infected macrophages using PrIntMap-R. (A) Average area over amino acid sequence of P08238 for control injections (orange) vs PO_4_ase treated samples (blue) searched in MSFragger without a variable phosphorylation mod. (B) Zoomed in version of A showing the region of interest. (C) Average area over amino acid sequence of P08238 for control injections (no PO_4_ase treatment) searched in MSFragger with a variable phosphorylation mod (blue) and without a variable phosphorylation mod (orange). (D) Area fold change plotted vs amino acid sequence for the same data as in panel C, with Uniprot annotated phosphosites designated by green lines. Coverage of P08238 was visualized with PrIntMap-R, comparing the results from the two searches.

The output plots can be readily interpreted and are mostly self-explanatory: They consist of amino acid sequence on the *x*-axis and appropriately selected peptide intensity metric on the *y*-axis, with the option for the *y*-axis to be represented on a linear or logarithmic scale. The plots are generated with the *plotly* package which allows for interaction.^23^ Users can zoom in and out on the plot, as well as hover the mouse over the data to see additional information. For example, hovering over an amino acid position can display all of the peptides that contributed to the intensity value at that position (Figure 2).

Plot options in PrIntMap-R that each show a different plot, are sorted as tabs by number of samples to be analyzed. *One sample* contains four possible views. The *Intensity trace* is the basic plot. *Stacked peptides* show all of the individual peptides that were mapped to the protein of interest in a stepwise fashion, colored by intensity. *Unique peptides* color each amino acid position by whether its sequence was mapped to any other protein in the proteome or not, and whether it was repeated within the protein of interest. *Sequence coverage* calculates what percentage of the amino acids in a protein were observed as part of an identified peptide. *Multiple samples* allow for the comparison of two or more different samples with the options to overlay traces, show the difference from a control sample, or the fold change from a control sample. *Two samples* produces the same plots as *multiple samples*, but additionally creates a peptide volcano plot showing differences in peptide intensity between two samples with significance and fold change. In addition to these tabs, there are options to show annotations on any of the plots described above, found in collapsable menus. *Annotation* allows for the highlighting of specific sequence features, such as N-glycosylation motifs or protease cut sites.^24^ There are several predefined annotation options, as well as the option for the user to define custom annotations. *PTMs* allow for the annotation of post-translational modifications (PTMs) that were identified during the database search. All other plot types described above can be annotated with PTMs if the peptide file is in the PEAKS or generic .csv format. Finally, there is an option to export the underlying data that generated each plot as a separate .csv file, so that users can easily create their own figures in the program of their choice. Additionally, each plot is available to export as a high resolution file in the .png format which is compatible with popular figure creation software, such as Adobe Illustrator.

### Data Analysis

Several use cases are presented here to demonstrate the utility of PrIntMap-R. The first example comes from injections of *Escherichia coli* β-galactosidase (βGal) digest and bovine serum albumin (BSA) digest analyzed on a Bruker timsTOF mass spectrometer per previously described methods.^25^ The next data set comes from phosphorylation analysis by Dreier et al., where bone marrow derived macrophages were infected with *Salmonella enterica*, followed by digestion, phosphopeptide enrichment, and enzymatic dephosphorylation.^26^ Raw MS files were downloaded from PRIDE project PXD007528.^27^ Data were searched using Fragpipe Version 18.0 and MSFragger version 3.5 with the “default” search workflow, along with label free quantification.^5,28^ One search included the *Phosphorylation (STY)* PTM, and another search was done without this PTM. Each injection was searched as its own sample, and replicates were combined using PrIntMap-R. Control samples (no dephosphorylation) were compared to phosphatase treated samples, and the different search strategies were also compared. Details on search parameters and the exact files used can be found in the Supporting Information.

The third data set comes from raw MS files gifted from the lab of Rebecca J. Whelan. These samples contain the proteome derived from Human Serum treated with PNGase F which removes N-linked glycans (PXD037921). These MS files were searched with the *De Novo* Assisted Database Search using PEAKS proteomics software Online X build 1.4.2020-10-21_171258, using the default search parameters.^7^ Each injection was searched as a separate sample, followed by the combination of replicates with PrIntMap-R. Control samples without PNGase F treatment were compared with PNGase F treated samples. See the Supporting Information for detailed search parameters and files used.

The final data set comes from a nonspecific digestion of histone proteins by Mullahoo et al. using a combination of protease type XIII and pepsin, for the purpose of performing hydrogen–deuterium exchange MS (HDX-MS).^29^ MS files were downloaded from the MassIVE (Mass Spectrometry Interactive Virtual Environment) project.^30^ The files used in this analysis were from the optimization portion of the Mullahoo et al. publication, where different digestion flow rates and times were evaluated. The MS files were searched using *De Novo* Assisted Database Search using PEAKS proteomics software Online X build 1.4.2020-10-21_171258, using the default search parameters but with “Unspecific” digestion specified. See the Supporting Information for detailed search parameters and files used.

For all of the analysis described above, the human reference proteome downloaded from Uniprot on Aug 23, 2022 was used.^31^ The search result files from the MSFragger and PEAKS searches were downloaded and used for analysis with PrIntMap-R. These data sets are available to download under the “examples” tab in PrIntMap-R. All figures created with PrIntMap-R version 0.1.x. The legends and titles of the plots created by PrIntMap-R were left in the figures of this paper for clarity.

## Results and Discussion

### Basic Analysis

The basic plot produced by PrIntMap-R is the amino acid number of a protein of interest on the *x*-axis, and the measured intensity (LFQ area, TIC intensity, or PSMs) on the *y*-axis. To demonstrate these basic plots, an injection of *E. coli* β-galactosidase (βGal) digest and BSA digest were each searched against a human proteome database containing a custom entry, which contained a virtual fusion protein made up of the entire βGal sequence appended to the entire BSA sequence. The LFQ intensity at each amino acid position of this fusion protein for the BGal injection and the BSA injection can be shown in SI Figure 1, showing detected signal in the N-terminal region for the βgal sample, and signal in the C-terminal region of the BSA sample. This simple use case illustrates the potential for PrIntMap-R to be used as a coverage visualization tool for proteoforms where some domains or splice variants are expressed at higher levels than others. One common application of this is mapping the approximate domains of a processed protein extracted and digested from sodium dodecyl sulfate polyacrylamide gel electrophoresis.

### Phosphorylation Analysis

To demonstrate the sample comparison features of PrIntMap-R, phosphopeptide-enriched macrophage MS files were analyzed with MSFragger database searches. Two sets of samples were compared: those that had been treated with alkaline phosphatase (PO_4_ase) to remove phosphorylation, and control samples that had not. First, a database search was performed without looking for phosphorylation PTMs, and the coverage of heat shock protein HSP 90-beta (P08238, a protein with known phosphorylation sites) was analyzed.^32,33^Figure 2A shows the LFQ quantified coverage of HSP90 across the amino acid sequence, comparing the PO_4_ase sample with the control sample as visualized with PrIntMap-R. Several peptides were identified only in the control, but importantly there are two peptide regions between residues 210 and 270 that show a several fold increase in LFQ intensity with PO_4_ase treatment. As is discussed by Dreier et al., this is likely due to the increased ionization efficiency of phosphopeptides with the removal of the suppression from negatively charged phosphates. PrIntMap-R provides an easy and quick visualization tool to see this difference in any protein of interest. Figure 2B shows a zoomed in view of the region of interest, which is easily achieved in PrIntMap-R using the built-in *plotly* manipulation tools.

Another analysis that can be visualized with the same data set involves comparing database searches. The control injections (no PO_4_ase treatment) were searched in MSFragger, both with and without a variable phosphorylation PTM included in the search parameters. The LFQ intensity across the amino acid sequence of P08238 was visualized with PrIntMap-R. From this comparison regions which gain in intensity can thus be linked with the difference in search parameters, in this case the addition of the phosphorylation PTM (Figure 2C). There were three obvious regions where more LFQ area was detected when searching with the phosphorylation PTM (Amino acids 220–242; 248–266; 439–448), lending support to the conclusion that these regions contain sites of phosphorylation. A zero value for the 439–448 peptide could potentially also occur in cases where data-dependent acquisition failed to fragment a mass (missing value). Performing a search with “missing-values” or similar enabled should reduce this effect if it is a concern. Additionally, these data can be visualized as a fold change plot (Figure 2D), and the annotated phosphosites from Uniprot (green annotations) line up well with the three identified spikes in LFQ intensity. In this way, PrIntMap-R can be used to visualize regions where PO_4_ase successfully cleaved phosphate modifications, and the visualizations can also be used to compare the results from different database search strategies. In this case, MSFragger successfully identified phosphopeptides when looking for the mass shift caused by phosphorylation.

### Glycosylation Analysis

**Figure 3:**
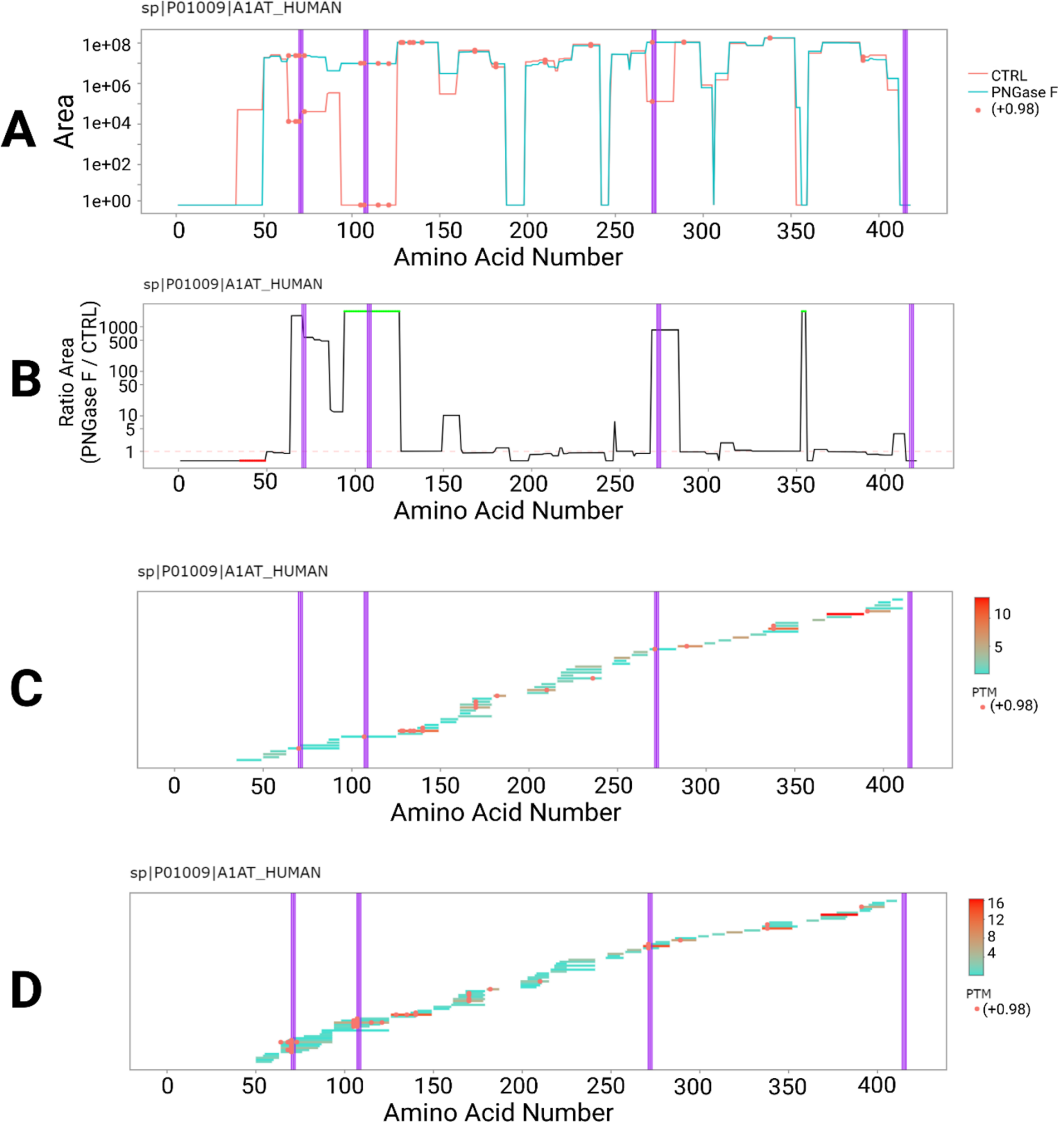
Deglycosylation Analysis of Alpha-1-Antitrypsin (P01009) from human serum using PrIntMap-R. (A–D) Purple lines show location of N-glyco motif (N-X-S/T) and (A,C,D) orange dots show location of deamidation PTM. (A) Average detected area across amino acid sequence for a control sample (9 injections, orange), and a deglycosylated sample (9 injections, blue). (B) Average area fold changes (deglycosylated/control) across the amino acid sequence, with green dots indicating positive infinite values and red dots indicating negative infinite values. (C) Individual detected peptides in the control sample and (D) deglycosylated sample mapped to their position on the amino acid sequence. Color indicates the average number of PSMs for each peptide. Data from PXD097921.

To illustrate the utility of annotating PTMs within PrIntMap-R, results from PNGase F deglycosylated human serum samples were compared against control human serum samples to look for increased coverage at sites of glycosylation (Figure 3). N-glycans are canonically found at an N-glycan motif composed of N-X-S/T, where the asparagine residue is the site of glycosylation, X is any amino acid except for proline, and the third amino acid is either serine or threonine.^24^ Deglycosylation with PNGase F results in the full cleavage of an N-glycan, along with the conversion of an asparagine residue to aspartic acid, essentially a deamidation. Because glycans significantly affect the mass of peptides, we would expect to not identify glycosylated peptides using a standard database search, whereas we can readily detect their deglycosylated counterparts by searching with a deamidation PTM. The combination of the N-glycosylation motif, a + 0.98 Da mass shift (deamidation), and higher intensity in the deglycosylated sample is evidence of a potential glycosite. The visualization of all three of these features is fast and easily performed using PrIntMap-R (Figure 3). Alpha-1-antitrypsin (P01009) was chosen to illustrate the glycosite identification for a representative protein identified from a tryptic digest of a complex human proteome sample. In Figure 3A–D, the N-glyco motif is annotated as vertical purple lines highlighting the three amino acids that make up the motif, a feature of the “Annotation” tab in PrIntMap-R. Figure 3A compares the average detected area across the amino acid sequence of P01009 between control samples (orange) and deglycosylated samples (blue), with three of the glyco-motifs lining up well with regions where the deglycosylated area is larger than the control area. Deamidation modifications can be seen at these same three positions (Asn70, Asn107, and Asn271), further supporting the conclusion that N-linked glycans were present at these positions in the original sample. Another method of looking at the same data can be seen in Figure 3B, where the fold change between the samples is plotted, resulting in spikes at the three previously mentioned positions. For fold-change plots in PrIntMap-R, it is necessary to visualize infinite and negative infinite values. These occur when intensity is observed in one sample, and not in the other. For ease of visualization, negative infinity-values are displayed at *y* = 0, but with red highlights (Seen between amino acids 40–50 in Figure 3B). Positive infinity values are shown at 1.25× the maximum *y*-value in the plot, and highlighted in green (seen between amino acids 90–120 in Figure 3B). In Figure 3C the individual peptides observed in the control sample can be compared to those seen in the deglycosylated sample (Figure 3D), showing that there are several additional versions of peptides covering the positions of the glycosites detected after deglycosylation, a feature of the *stacked peptides* plot in PrIntMap-R.

PrIntMap-R also can produce a volcano plot comparing peptide abundance and significance between two samples, which for this example are control and deglycosylated injections (SI Figure 2A). In this case, there are many peptides from P01009 that are not significantly increased or decreased, but there are also many more that were only detected in the deglycosylated sample (shown in the top right corner of the plot). PrIntMap-R displays “infinite fold change” (peptides that were present in one sample but not the other) in these boxes. PrIntMap-R also allows the user to select whether or not to display “compromised values”. These are peptides that have enough observations to calculate significance in one sample, but not enough observations to calculate significance in the other sample. Depending on the application, users may or may not want to include these peptides in their analysis and visualization. SI Figure 2B shows an example of the additional data that can be found by hovering the mouse over each data point in the volcano plot. Volcano plots are common in proteomics to visualize fold-change and significance between proteins in different samples, but are less frequently used for peptide analysis. While the statistical rigor of this type of plot has not been explored in detail, we believe it is still useful to visually identify specific peptides that may be of interest, and the color coding within PrIntMap-R allows a user to see whether or not they map to the sequence of a protein of interest. Overall, the various tools available in PrIntMap-R allow for the visualization of PTMs, sequence motifs, and the direct comparison between two samples in a variety of ways, in this example by predicting the glycosylation sites at three positions on P01009.

### Histone Coverage Analysis

To demonstrate the usefulness of the stacked peptide plots and multiple sample comparison of PrIntMap-R, optimization experiments from nonspecific digests of H4 protein were analyzed.

**Figure 4:**
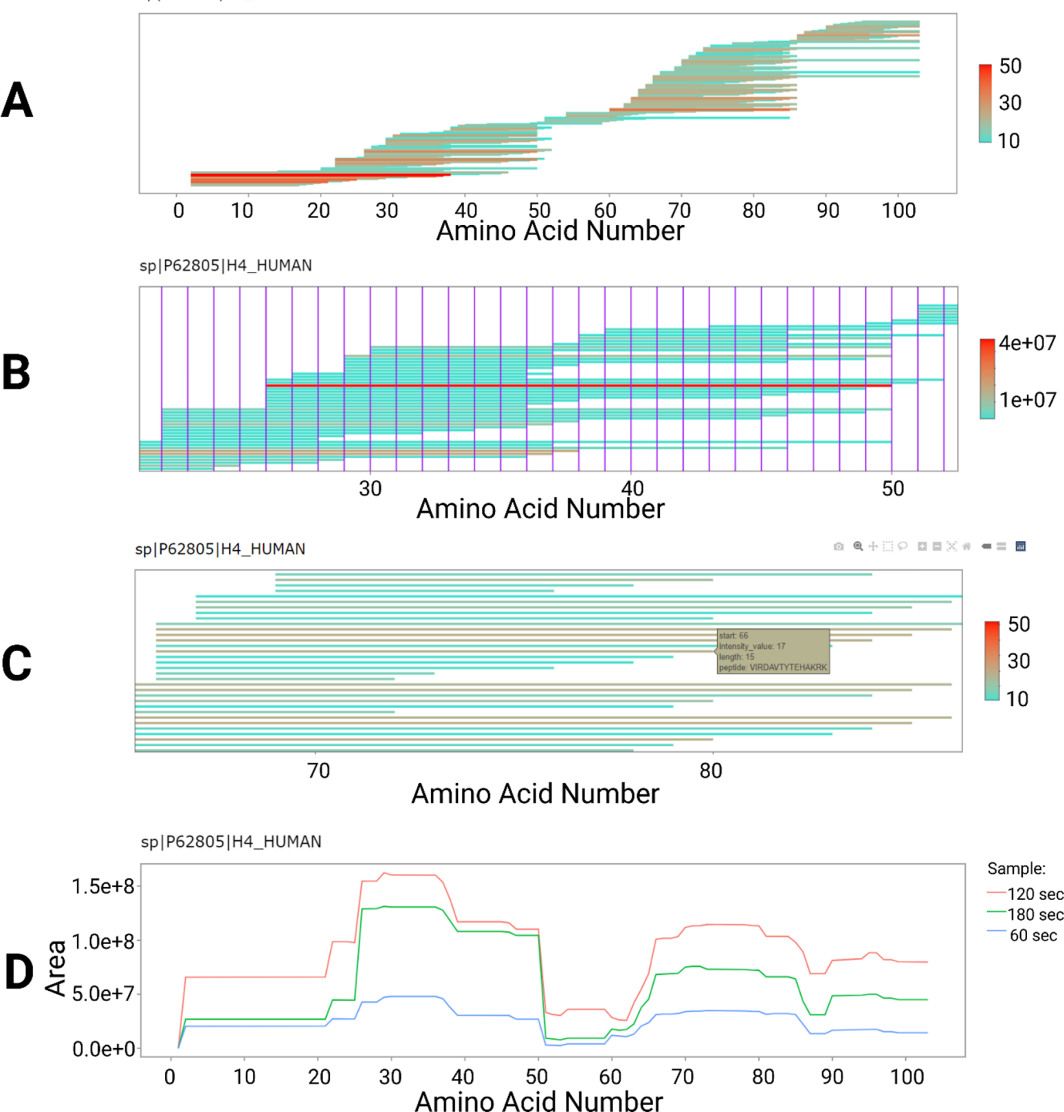
Peptide coverage analysis of H4 histone protein from nonspecific digestion using PrIntMap-R. (A) Peptides observed in optimization experiments mapped to the H4 amino acid sequence, with color based on the average number of PSMs. (B) Zoomed in view of the same plot, with color now based on average area, and each amino acid position marked with a purple line. (C) Another zoomed view of the same plot, color based on average PSMs, showing the pop-up window with additional peptide information. (D) Comparison of three experimental conditions for digestion times: orange, 120 s; green, 180 s; blue, 60 s; with observed area on the *y*-axis.

These digestions are intended to be used for HDX-MS, where coverage of the entire protein sequence, individual amino acid resolution of the proteases, and multiple peptide coverage of each amino acid position are all desirable outcomes.^29^Figure 4A shows the stacked peptide plot coverage of the H4 protein from all the optimization experiments. This type of plot is commonly published in HDX-MS papers, and PrIntMap-R easily generates this plot with coloration based on area, PSMs (as in this case), or other intensity metrics. This plot can also be annotated with identified PTMs and/or sequence specific annotations. Figure 4B shows the same plot, but demonstrates a zoomed in view, which is easily achieved in PrIntMap-R by highlighting a particular region of the plot. Additionally, each amino acid position has been annotated by a vertical purple line, which aids in the identification of which amino acids contain better or worse peptide coverage, and which amino acids tend to be on the terminal ends of peptides. Figure 4C shows another zoomed in view, with an example popup box with additional information about the peptide.

Lastly, PrIntMap-R provides an easy way to compare multiple samples, helpful for optimization experiments. In this case, three different digestion times were tested (60, 120, and 180 s), with different results. As shown in Figure 4D, the area detected across the entire H4 sequence was higher in the 120 s samples compared to the other conditions. Importantly, percent coverage of H4 based on the different experimental conditions is essentially identical (as is reported by many database search software), so the visualization with PrIntMap-R is helpful for quickly identifying optimal conditions. Other experimental conditions such as flow rate can be compared (SI Figure 3A), and many different samples can be compared at once as overlaid traces (9 different injections shown in SI Figure 3B), or as fold change from one specified injection (SI Figure 3C). The combination of the stacked peptide plots, annotations, and multiple sample comparison tools make PrIntMap-R a useful app for optimizing sample preparation experiments.

## Conclusion

Through the use cases demonstrated here, we have shown the utility of PrIntMap-R in visualizing protein coverage for the purposes of comparing one or multiple samples, comparing search strategies, hypothesizing modification sites, viewing depth of peptide coverage and protease resolution, and optimizing sample preparation. In addition to the plots shown here, there are features in PrIntMap-R to evaluate the uniqueness of identified peptides (similar to SI Figure 1AB, 2C) which could be useful to identify whether peptides of high intensity originate from a protein of interest. Another potential use for PrIntMap-R is as a protein domain visualization tool. For example, to visualize the expression of splice variants where the intensity of protein domains varies in different experimental conditions, even if the protein abundance overall is equivalent. Any numerical value can be substituted for “Intensity”; we include a template within the program which will allow laboratories to generate their own use-cases for displaying other molecular data including turnover rate, or coordinance with sequencing data. PrIntMap-R is continually being improved and updated. Future features will include the option to take abundance data from other sources such as iTraq/TMT and DIA (DIA-NN) data.^34−37^

## Data Availability

PrIntMap-R is available online (https://championlab.shinyapps.io/printmap-r/) and as a downloadable package that can be run locally (https://github.com/Champion-Lab/PrIntMap-R). Instructions for local installation are found at the GitHub. A mirror is available at Zenodoo (https://zenodo.org/record/7324824#.Y5FLFXbMKUk).

## Supporting Information Available

Additional uses of PrintMap-R for fusion-protein characterization, glycan analysis, and HDX optimization as well as search parameters, sample information, and supplemental figures (PDF)
