# Supplementary material for: PrIntMap-R: An Online Application for Intraprotein Intensity and Peptide Visualization from Bottom-Up Proteomics
Source: J Proteome Res. 2023 Jan 18;22(2):432–41. doi: 10.1021/acs.jproteome.2c00606 (PMC9904286; doi:10.1021/acs.jproteome.2c00606)
Supplement: Supplementary file 1 — pr2c00606_si_001.pdf [file pr2c00606_si_001.pdf]

Supporting Information for:

PrIntMap-R: an Online Application for Intraprotein Intensity and Peptide Visualization from Bottom-Up Proteomics

Simon D. Weaver[1,2], Christine M. DeRosa[1], Sadie R. Schultz[1], and Matthew M. Champion*[1,2]

[1]Department of Chemistry and Biochemistry, University of Notre Dame, Notre Dame, IN, USA 46556
[2]Integrated Biomedical Sciences Graduate Program, University of Notre Dame, Notre Dame, IN, USA 46556

*Corresponding author:
Address to whom correspondence should be addressed:
mchampio@nd.edu

Table of Contents

1.) SI Figure 1: Analysis of example fusion protein Beta-Galactosidase/Bovine Serum Albumin.
2.) SI Figure 2: Additional PrIntMap-R features for glycosylation analysis of P01009.
3.) SI Figure 3: Multi-sample comparison in PrIntMap-R for optimization of HDX-MS experiments.
4.) Supplementary Methods and Search Parameters
    a.) glyco analysis in PEAKS
    b.) HDX-MS optimization in PEAKS
    c.) Phosphopeptides in MSFragger
    d.) Example Fusion Protein in PEAKS

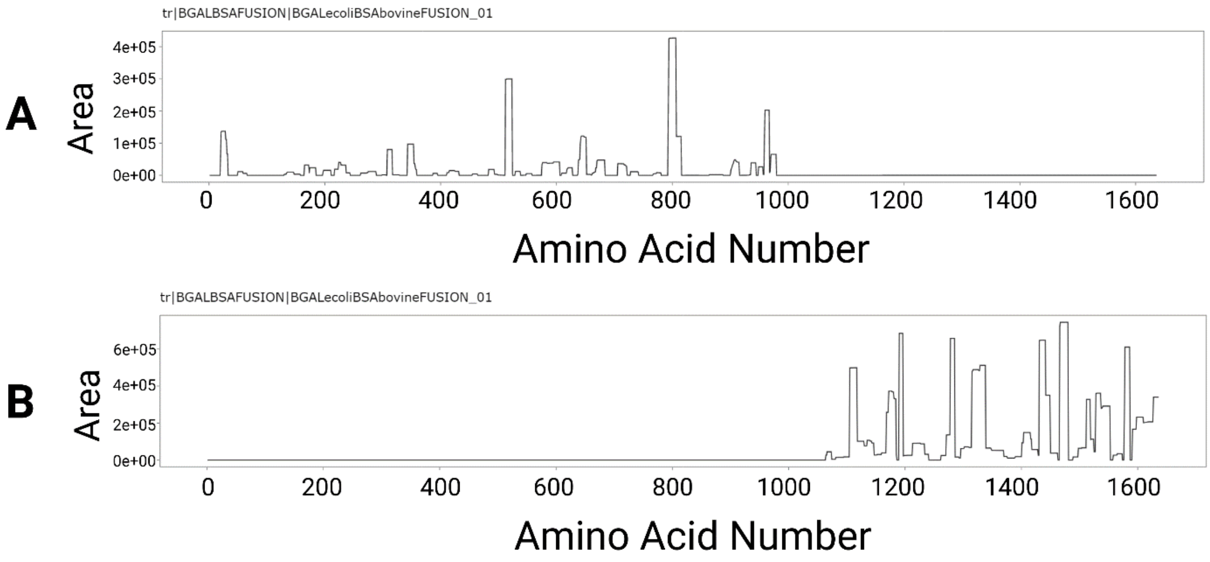

SI Figure 1: Analysis of example fusion protein β-Galactosidase/Bovine Serum Albumin. *Digestion of (**A**) E. coli β-Galactosidase (BGal) and (**B**) Bovine Serum Albumin (BSA) each searched against a database containing a fusion protein containing the β Gal amino acid sequence at the N-terminus and the BSA amino acid sequence at the C-terminus. PrIntMap-R was used to map the resulting LFQ Area onto the amino acid sequence of this in-silico artificial fusion protein, showing the difference in intensity at different domains.*

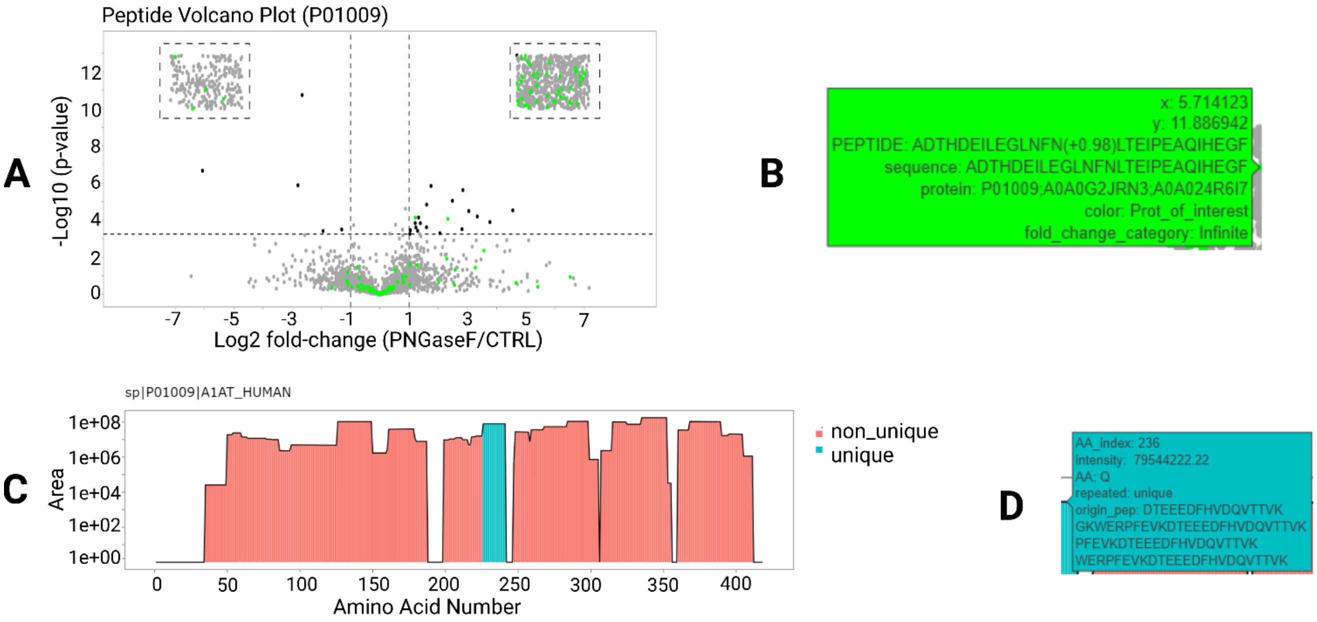

SI Figure 2: Additional PrIntMap-R features for glycosylation analysis of P01009. *(**A**) Volcano plot showing all the peptides identified. Log_2 fold change is based on Deglycosylated / Control. Infinite values shown in dotted boxes at the top corners of the plot. Green points are peptides that were mapped to the protein of interest (P01009), black points are peptides that fall above the significance and fold change thresholds, and gray points are peptides that do not. (**B**) Example of popup with extra information when 'mousing' over one of the points in the volcano plot. (**C**) Unique peptide plot, pink regions were identified peptides that mapped to elsewhere in the proteome database, while blue regions were unique to P01009. (**D**) Example of popup with extra information when 'mousing' over one of the points in the unique peptide plot. Data from PXD09721.*

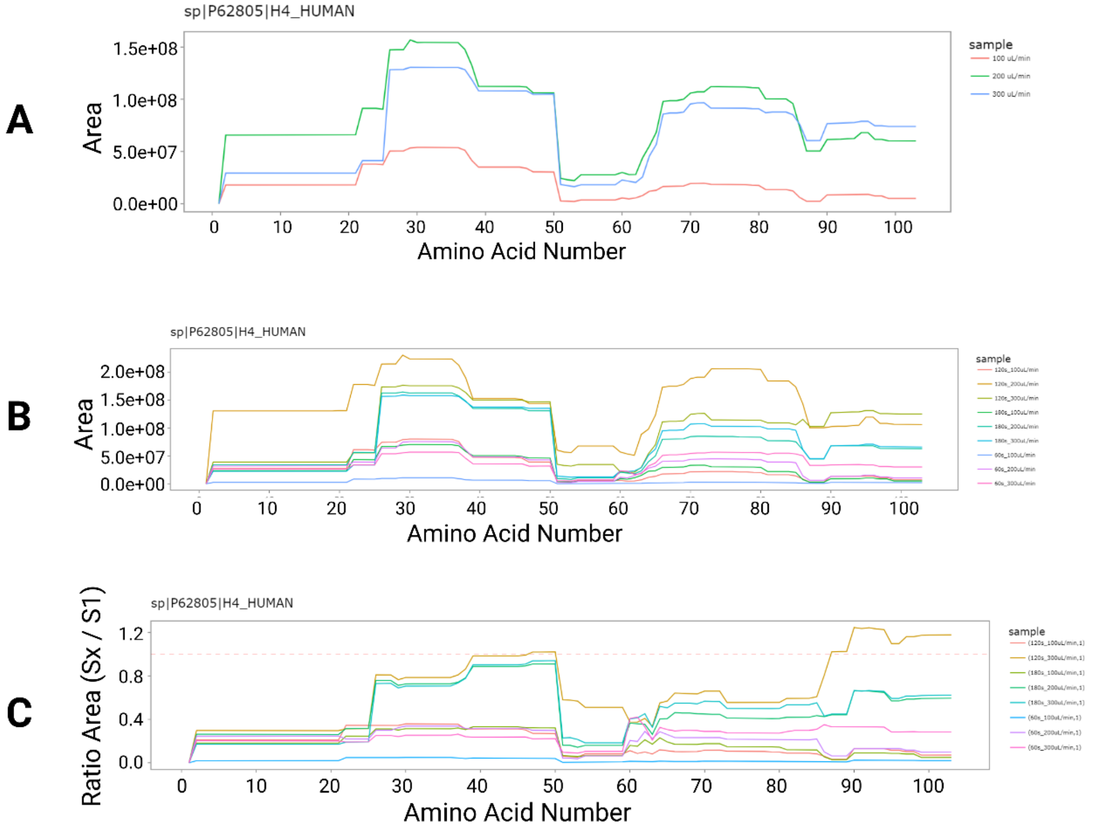

SI Figure 3: Multi-sample comparison in PrIntMap-R for optimization of HDX-MS experiments. *(**A**) Comparison of three experimental conditions for flow rate: Orange: 100 µL/min, Green: 200 µL/min, Blue: 300 µL/min, with observed area on the y-axis. (**B**) Nine-sample comparison for each experimental condition (flow rate and time), with observed area on the y-axis. (**C**) Nine-sample area fold change comparison for each experimental condition, where the fold change is based on the 200 µL/min, 120 second sample.*

Supplementary Methods and Search Parameters:

a) Search parameters for glyco analysis in PEAKS:

Precursor Mass Error Tolerance: 10 ppm
Fragment Mass Error Tolerance: 0.02 Da
Missed Cleavages: 3
Taxonomy: all species
PSM FDR: ≤ 1.00%
Proteins Unique Peptides: 1
Max variable PTM per peptide: 3
Enzyme: Trypsin
Protiens –10LgP: ≥20
Digest Mode: SemiSpecific
Peptide length: 6-45 amino acids
Fixed Mods: Cabamidomethylation
Variable Mods: Deamidation (NQ), Oxidation (M), Pyro-glu from E, Pyro-glu from Q, Sodium Adduct

Samples: Three bio replicates, each with three technical replicates (injections) for both control and PNGase F treated samples were analyzed. Files were a gift from Rebecca J. Whelan, and can be found in PRIDE database with identifier PXD037921

b) Search parameters for HX-MS optimization in PEAKS

Precursor Mass Error Tolerance: 10 ppm
Fragment Mass Error Tolerance: 0.02 Da
Missed Cleavages: 3
Taxonomy: all species
PSM FDR: ≤ 1.00%
Proteins Unique Peptides: 1
Max variable PTM per peptide: 3
Enzyme: Specified by each sample
Protiens –10LgP: ≥20
Digest Mode: Unspecific
Peptide length: 6-45 amino acids

Samples: One injection for each of 9 conditions was analyzed. Each condition consisted of one of the following flow rates (100, 200, or 300 µL/min) and one of the following digestion times (60, 120, or 180 seconds) with all of the possible permutations analyzed. For comparisons for only one of the variables, the three injections with the same condition for that variable were combined and averaged in PrIntMap-R. Files downloaded from:

https://massive.ucsd.edu/ProteoSAFe/dataset.jsp?task=dffde7a5ee724c25b554400024fe8cf8
Files used were:

MSV000083870 H4_Digestion_Optimization_ETD/H4_ETD_z2_8_1ug_NN.raw Where NN is:
Dig60s_100ulmin_01
Dig60s_200ulmin_01
Dig60s_300ulmin_01
Dig120s_100ulmin_01
Dig120s_200ulmin_01
Dig120s_300ulmin_01
Dig180s_100ulmin_01
Dig180s_200ulmin_01
Dig180s_300ulmin_01

c) Search parameters for Phosphopeptides in MSFragger

With Phosphorylation PTM (For search without Phospho PTM, identical parameters were used, but the variable modification:*variable_mod_03 = 79.966330 STY 3* was not included. highlighted in the parameters below.)

database_name = E:\Active_Data\Simon\Printmap_use_cases\Figure_Creation_2022_08_23_Phosphopeptides\2022-08-23-decoys-HUMAN_uniprot-download_true_format_fasta_query__28proteome_3AUP000005640_2-2022.08.23-18.56.13.61.fasta.fas # Path to the protein database file in FASTA format.
num_threads = 26 # Number of CPU threads to use.

precursor_mass_lower = -20 # Lower bound of the precursor mass window.
precursor_mass_upper = 20 # Upper bound of the precursor mass window.
precursor_mass_units = 1 # Precursor mass tolerance units (0 for Da, 1 for ppm).
data_type = 0 # Data type (0 for DDA, 1 for DIA, 2 for gas-phase fractionation DIA).
precursor_true_tolerance = 20 # True precursor mass tolerance (window is +/- this value).
precursor_true_units = 1 # True precursor mass tolerance units (0 for Da, 1 for ppm).
fragment_mass_tolerance = 20 # Fragment mass tolerance (window is +/- this value).
fragment_mass_units = 1 # Fragment mass tolerance units (0 for Da, 1 for ppm).
calibrate_mass = 2 # Perform mass calibration (0 for OFF, 1 for ON, 2 for ON and find optimal parameters).
use_all_mods_in_first_search = 0 # Use all variable modifications in first search (0 for No, 1 for Yes).
decoy_prefix = rev_ # Prefix of the decoy protein entries. Used for parameter optimization only.

deisotope = 1 # Perform deisotoping or not (0=no, 1=yes and assume singleton peaks single charged, 2=yes and assume singleton peaks single or double charged).
deneutralloss = 1 # Perform deneutrallossing or not (0=no, 1=yes).
isotope_error = 0/1/2/3 # Also search for MS/MS events triggered on specified isotopic peaks.
mass_offsets = 0 # Creates multiple precursor tolerance windows with specified mass offsets.
precursor_mass_mode = selected # One of isolated/selected/corrected.

remove_precursor_peak = 1 # Remove precursor peaks from tandem mass spectra. 0 = not remove; 1 = remove the peak with precursor charge; 2 = remove the peaks with all charge states (only for DDA mode).
remove_precursor_range = -1.500000,1.500000 # m/z range in removing precursor peaks. Only for DDA mode. Unit: Th.
intensity_transform = 0 # Transform peaks intensities with sqrt root. 0 = not transform; 1 = transform using sqrt root.
activation_types = all # Filter to only search scans of provided activation type(s). Allowed: All, HCD, CID, ETD, ECD.

write_calibrated_mgf = 0 # Write calibrated MS2 scan to a MGF file (0 for No, 1 for Yes).
mass_diff_to_variable_mod = 0 # Put mass diff as a variable modification. 0 for no; 1 for yes and remove delta mass; 2 for yes and keep delta mass.

localize_delta_mass = 0 # Include fragment ions mass-shifted by unknown modifications (recommended for open and mass offset searches) (0 for OFF, 1 for ON).
delta_mass_exclude_ranges = (-1.5,3.5) # Exclude mass range for shifted ions searching.
fragment_ion_series = b,y # Ion series used in search, specify any of a,b,c,x,y,z,Y,b-18,y-18 (comma separated).

ion_series_definitions = *# User defined ion series. Example: "b* N -17.026548;b0 N -18.010565".*

labile_search_mode = off *# type of search (nglycan, labile, or off). Off means non-labile/typical search.*
restrict_deltamass_to = all *# Specify amino acids on which delta masses (mass offsets or search modifications) can occur. Allowed values are single letter codes (e.g. ACD) and '-', must be capitalized. Use 'all' to allow any amino acid.*
diagnostic_intensity_filter = 0 *# [nglycan/labile search_mode only]. Minimum relative intensity for SUM of all detected oxonium ions to achieve for spectrum to contain diagnostic fragment evidence. Calculated relative to spectrum base peak. 0 <= value.*
Y_type_masses = *# [nglycan/labile search_mode only]. Specify fragments of labile mods that are commonly retained on intact peptides (e.g. Y ions for glycans). Only used if 'Y' is included in fragment_ion_series.*
diagnostic_fragments = *# [nglycan/labile search_mode only]. Specify diagnostic fragments of labile mods that appear in the low m/z region. Only used if diagnostic_intensity_filter > 0.*
remainder_fragment_masses = *# [labile search_mode only] List of possible remainder fragment ions to consider. Remainder masses are partial modification masses left on b/y ions after fragmentation.*

```
search_enzyme_name_1 = stricttrypsin          # Name of the first enzyme.
search_enzyme_cut_1 = KR                      # First enzyme's cutting amino acid.
search_enzyme_nocut_1 =                       # First enzyme's protecting amino acid.
search_enzyme_sense_1 = C                     # First enzyme's cutting terminal.
allowed_missed_cleavage_1 = 2                 # First enzyme's allowed number of missed cleavages per peptide. Maximum value is 5.

search_enzyme_name_2 = null                   # Name of the second enzyme.
search_enzyme_cut_2 =                         # Second enzyme's cutting amino acid.
search_enzyme_nocut_2 =                       # Second enzyme's protecting amino acid.
search_enzyme_sense_2 = C                     # Second enzyme's cutting terminal.
allowed_missed_cleavage_2 = 2                 # Second enzyme's allowed number of missed cleavages per peptide. Maximum value is 5.

num_enzyme_termini = 2                        # 0 for non-enzymatic, 1 for semi-enzymatic, and 2 for fully-enzymatic.

clip_nTerm_M = 1                              # Specifies the trimming of a protein N-terminal methionine as a variable modification (0 or 1).

# maximum of 16 mods - amino acid codes, * for any amino acid, [ and ] specifies protein termini, n and c specifies peptide termini
variable_mod_01 = 15.994900 M 3
variable_mod_02 = 42.010600 [^ 1
variable_mod_03 = 79.966330 STY 3
variable_mod_04 = -17.026500 nQnC 1
variable_mod_05 = -18.010600 nE 1
# variable_mod_06 = 4.025107 K 2
# variable_mod_07 = 6.020129 R 2
# variable_mod_08 = 8.014199 K 2
# variable_mod_09 = 10.008269 R 2
# variable_mod_10 = 0.000000 site_10 1
# variable_mod_11 = 0.000000 site_11 1
# variable_mod_12 = 0.000000 site_12 1
# variable_mod_13 = 0.000000 site_13 1
# variable_mod_14 = 0.000000 site_14 1
# variable_mod_15 = 0.000000 site_15 1
# variable_mod_16 = 0.000000 site_16 1

allow_multiple_variable_mods_on_residue = 0
max_variable_mods_per_peptide = 3             # Maximum total number of variable modifications per peptide.
max_variable_mods_combinations = 5000         # Maximum number of modified forms allowed for each peptide (up to 65534).

output_format = pepXML_pin                    # File format of output files (tsv, pin, pepxml, tsv_pin, tsv_pepxml, pepxml_pin, or tsv_pepxml_pin).
output_report_topN = 1                        # Reports top N PSMs per input spectrum.
output_max_expect = 50                        # Suppresses reporting of PSM if top hit has expectation value greater than this threshold.
report_alternative_proteins = 1               # Report alternative proteins for peptides that are found in multiple proteins (0 for no, 1 for yes).

precursor_charge = 1 4                        # Assumed range of potential precursor charge states. Only relevant when override_charge is set to 1.
override_charge = 0                           # Ignores precursor charge and uses charge state specified in precursor_charge range (0 or 1).

digest_min_length = 7                         # Minimum length of peptides to be generated during in-silico digestion.
digest_max_length = 50                        # Maximum length of peptides to be generated during in-silico digestion.
digest_mass_range = 500.0 5000.0              # Mass range of peptides to be generated during in-silico digestion in Daltons.
max_fragment_charge = 2                       # Maximum charge state for theoretical fragments to match (1-4).

track_zero_topN = 0                           # Track top N unmodified peptide results separately from main results internally for boosting features.
zero_bin_accept_expect = 0                    # Ranks a zero-bin hit above all non-zero-bin hit if it has expectation less than this value.
zero_bin_mult_expect = 1                      # Multiplies expect value of PSMs in the zero-bin during  results ordering (set to less than 1 for boosting).
add_topN_complementary = 0                    # Inserts complementary ions corresponding to the top N most intense fragments in each experimental spectra.
```

check_spectral_files = 1 *# Checking spectral files before searching.*
minimum_peaks = 15 *# Minimum number of peaks in experimental spectrum for matching.*
use_topN_peaks = 150 *# Pre-process experimental spectrum to only use top N peaks.*
min_fragments_modelling = 2 *# Minimum number of matched peaks in PSM for inclusion in statistical modeling.*
min_matched_fragments = 4 *# Minimum number of matched peaks for PSM to be reported.*
min_sequence_matches = 2 *# [nglycan/labile search_mode only] Minimum number of sequence-specific (not Y) ions to record a match.*
minimum_ratio = 0.01 *# Filters out all peaks in experimental spectrum less intense than this multiple of the base peak intensity.*
clear_mz_range = 0.0 0.0 *# Removes peaks in this m/z range prior to matching.*

add_Cterm_peptide = 0.000000
add_Nterm_peptide = 0.000000
add_Cterm_protein = 0.000000
add_Nterm_protein = 0.000000

add_G_glycine = 0.000000
add_A_alanine = 0.000000
add_S_serine = 0.000000
add_P_proline = 0.000000
add_V_valine = 0.000000
add_T_threonine = 0.000000
add_C_cysteine = 57.021460
add_L_leucine = 0.000000
add_I_isoleucine = 0.000000
add_N_asparagine = 0.000000
add_D_aspartic_acid = 0.000000
add_Q_glutamine = 0.000000
add_K_lysine = 0.000000
add_E_glutamic_acid = 0.000000
add_M_methionine = 0.000000
add_H_histidine = 0.000000
add_F_phenylalanine = 0.000000
add_R_arginine = 0.000000
add_Y_tyrosine = 0.000000
add_W_tryptophan = 0.000000
add_B_user_amino_acid = 0.000000
add_J_user_amino_acid = 0.000000
add_O_user_amino_acid = 0.000000
add_U_user_amino_acid = 0.000000
add_X_user_amino_acid = 0.000000
add_Z_user_amino_acid = 0.000000

Samples: Samples downloaded from: https://www.ebi.ac.uk/pride/archive/projects/PXD007528

No PO_4ase:

Raw File
001_RD
002_RD
003_RD
004_RD
005_RD
006_RD
007_RD
008_RD
009_RD
010_RD
011_RD
012_RD
013_RD
014_RD
015_RD
016_RD
017_RD
018_RD

With PO_4ase:

Raw File
001_RD1
002_RD1
003_RD1
004_RD1
005_RD1
006_RD1
007_RD1
008_RD1
009_RD1
010_RD1
011_RD1
012_RD1
013_RD1
014_RD1
015_RD1
016_RD1
017_RD1
018_RD1

There were multiple different *Salmonella* infection times included within these data, but for the purpose of this demonstration, all of the infection times were combined in PrIntMap-R after being searched individually in MSFragger, so that the variable investigated was the PO_4ase treatment.

d) BSA and Beta-Galactosidase Example Fusion Protein Search Parameters in PEAKS
Search Parameters:

Precursor Mass Error Tolerance: 20 ppm
Fragment Mass Error Tolerance: 0.05 Da

Missed Cleavages: 3
Taxonomy: all species
PSM FDR: ≤ 1.00%
Proteins Unique Peptides: 1
Max variable PTM per peptide: 3
Enzyme: Specified by each sample (set to Trypsin within the sample options)
Protiens –10LgP: ≥20
Digest Mode: SemiSpecific
Peptide length: 6-45 amino acids
Fixed Mods: Cabamidomethylation
Variable Mods: Deamidation (NQ), Oxidation (M), Pyro-glu from E, Pyro-glu from Q, Sodium Adduct
